# Preclinical evaluation of *Luffa operculata* Cogn. and its main active principle in the treatment of bacterial rhinosinusitis^☆^

**DOI:** 10.1016/j.bjorl.2016.11.004

**Published:** 2016-12-26

**Authors:** Leonardo Silva, Henrique Olival Costa, Flávia Coelho de Souza, Elaine Monteiro Cardoso Lopes, Suely Mitoi Ykko Ueda

**Affiliations:** a Santa Casa de São Paulo, São Paulo, SP, Brazil; b Santa Casa de São Paulo, Faculdade de Ciências Médicas, São Paulo, SP, Brazil; c Instituto de Ciências Avançadas em Otorrinolaringologia (ICAO), São Paulo, SP, Brazil; d Universidade 9 de Julho (UNINOVE), São Paulo, SP, Brazil

**Keywords:** Sinusitis, Therapeutics, *Luffa*, Microbiology, *Streptococcus pyogenes*, Sinusite, Terapêutica, *Luffa*, Microbiologia, *Streptococcus pyogenes*

## Abstract

**Introduction:**

The prevalence of rhinosinusitis is quite high. Despite the widespread use of antibiotics for rhinosinusitis, there are other forms of treatment, including phytotherapy. One of the most widely used herbal medicines for treatment of rhinosinusitis is *Luffa operculata*.

**Objective:**

This study aimed to evaluate the efficacy of topical nasal solution of the aqueous extract of *L. operculata*, determining the toxicity to its use and identifying the active principles presented in the aqueous extract. The secondary objective was to evaluate the action of active principles on bacteria commonly involved in acute rhino sinusitis.

**Methods:**

The study was conducted in experimental model of sinusitis. Three different concentrations of *L. operculata* were used as local treatment of rhino sinusitis. The results were compared with those observed in control groups that received nasal saline solution. Histological examination of the liver, kidney, spleen, myocardium, brain and lungs of all animals evaluated the toxicity of *L. operculata*. The aqueous extract used was subjected to chromatographic analysis and an active principle was isolated and tested for in vitro inhibition of bacterial colonies usually found in rhino sinusitis.

**Results:**

Intranasal treatment of sinusitis with *L. operculata* showed better clinical evolution than control group. Statistically significant difference (*p* > 0.10) between the treated group and the control group was observed in the histologic evaluation for inflammatory pattern. The aqueous extract of *L. operculata* used presented a predominance of 2,3-dicafeoilglicaric acid, a substance not yet described in the literature. There was a significant difference in bacterial growth of *Streptococcus pyogenes* on blood-agar plates when under the influence of both the aqueous extract and the active substance.

**Conclusion:**

Topical nasal solution of the aqueous extract of *L. operculata* is effective compared to the application of saline solution for the treatment of bacterial rhinosinusitis in an experimental model. *L. operculata* determined in vitro inhibition of growth of *S. pyogenes*.

## Resumo

**Introdução:**

A prevalência de rinossinusite (RS) é bastante alta. Apesar do uso generalizado de antibióticos para RS, existem outras formas de tratamento, incluindo a fitoterapia. Uma das ervas medicinais mais utilizadas no tratamento da RS é a *Luffa operculata*.

**Objetivo:**

Esse estudo teve como objetivo avaliar a eficácia da solução tópica nasal do extrato aquoso de *Luffa operculata*, determinando a toxicidade ao seu uso e identificando os princípios ativos apresentados no extrato aquoso. O objetivo secundário foi avaliar a ação dos princípios ativos sobre as bactérias comumente envolvidas na RS aguda.

**Método:**

O estudo foi realizado em modelo experimental de sinusite. Utilizaram-se três concentrações diferentes de *Luffa operculata* como tratamento local de RS. Os resultados foram comparados com os observados em grupos de controle que receberam solução salina nasal. O exame histológico do fígado, rim, baço, miocárdio, cérebro e pulmões de todos os animais avaliou a toxicidade de *Luffa operculata*. O extrato aquoso utilizado foi submetido à análise cromatográfica e um princípio ativo foi isolado e testado para inibição in vitro de colônias bacterianas normalmente encontradas em RS.

**Resultados:**

O tratamento intranasal da sinusite com *Luffa operculata* mostrou melhor evolução clínica do que o do grupo controle. Foi observada diferença estatisticamente significante (p> 0.10) entre o grupo tratado e o grupo controle na avaliação histológica do padrão inflamatório. O extrato aquoso de *Luffa operculata* utilizado apresentou predominância do ácido 2,3-dicafeoilglicarico, substância ainda não descrita na literatura. Houve uma diferença significativa no crescimento bacteriano de *Streptococcus pyogenes* em placas de ágar-sangue quando sob a influência tanto do extrato aquoso quanto da substância ativa.

**Conclusão:**

A solução tópica nasal do extrato aquoso de *Luffa operculata* é eficaz em comparação com a aplicação de solução salina para o tratamento de RS bacteriana em um modelo experimental. *Luffa operculata* determinou a inibição in vitro do crescimento de *Streptococcus pyogenes*.

## Introduction

Acute RS is one of the commonest diagnoses in primary care, and its management has significant implications for both public health and costs.^1^ It is estimated that children have 7–10 common colds each year. The estimated frequency for adults is 2–5 episodes/year.^1^ About 0.5%–2% of these common colds result in acute bacterial RS.^2^ Sinusitis affects 1 in 7 adults in the United States; resulting in about 31 million individuals diagnosed each year.^3^

Data obtained in 2002 indicate that RS account for 9% of the prescribed antibiotics to children and 21% of prescribed antibiotics for adults, what makes it the fifth most common disease for which this type of medication is prescribed in the USA.^4^

Despite the widespread use of systemic antibiotics for sinusitis, there are many other forms of treatment, comprising several medications for systemic and local use. Systemic corticosteroids and Nonsteroidal Anti-Inflammatory Drug (NSAIDs), antihistamines, systemic and topical decongestants, anti-leukotrienes and local antiseptics, are employed for treatment of RS.^5^ Herbal medicine is also widely used by the population,^5^ although there are scarce no controlled experiments in the literature showing its effectiveness.^2^, ^6^, ^7^

Among the advantages of using herbal medicine are the wide acceptance of herbal and medicinal plants by the population due to cultural factors and the belief that been “natural”, present fewer adverse effects.^8^ The low cost and its abundance in tropical countries are other factors.^7^

One of the most widely used phytotherapics to treat RS in Brazil is the *Luffa operculata* used in preparations for nasal use.^9^

In a survey conducted in one popular market of Brazil, 86% (13 of 15) of plant vendors recommended *L. operculata* for treatment of RS.^10^

Chemical analysis of *L. operculata* shows that it has among its components glycosides, saponins, resin, free sterols, aliphatic esters, quinones, organic acids and phenols, and it does not contain tannins and flavonoids. In the resin are found elasterin A, B and D and cucurbitacines isocucurbitacin B.^9^

Despite the widespread use of *L. operculata*, there are few studies that prove its therapeutic value for sinusitis.^5^

The objective of this study is to evaluate the efficacy of topical nasal solution of the aqueous extract of *L. operculata*, determining the toxicity to its use and identifying the active principles presented in the aqueous extract. The secondary objective was to evaluate the action of *L. operculata* active principles on bacteria commonly involved in acute RS.

## Methods

### Induction of RS in the animal model

The study was submitted to the ethics committee in research and approved under number 2011-3. A veterinarian accompanied all procedures performed on animals. Institutional guidelines regarding animal experimentation were followed.

One hundred eighty adult white New Zealand rabbits, of both genders, weighing approximately 2500 g at the beginning of the experiment were used. Throughout the study, animals were confined in individual cages suitable for race and weight.

The animals were divided into 3 groups. One group was followed for therapeutic evaluation of *L. operculata*. This group was followed for 3 different periods of time. Another group was untreated with *L. operculata* (control group) and finally one group received *L. operculata* to assess its toxicity. Therefore, each group had twenty animals for each follow-up time. Thus we evaluated 20 animals for therapeutic group (*n* = 60), 60 for the control group and 60 for the toxicity group.

The rabbits were submitted to surgical procedures under general anesthesia in order to generate a nasal inflammatory process, similar to acute infectious RS. Initially a porous sponge of polyvinyl measuring 3.0 cm × 0.5 cm × 0.3 cm was sterilized in ethylene oxide and then introduced in one nasal cavity of each animal.

One mL of a solution composed of 0.8 mL of the animal blood and 0.2 mL of streptococcal and staphylococcal toxoid (Toxoidepot^®^), was injected percutaneous in maxillary antrum on the same side were the sponge was introduced. The sponges were maintained in the nasal cavity of each animal for a period of ten days. After this period the sponges were removed and the treatment period began. When the sponge was removed from the nasal cavity and before the start of the treatment each animal had the secretion of the nasal cavity collected by swab (Cuturet^®^). Samples of sinus secretions were smeared on blood-agar culture media and chocolate-agar (Probac do Brasil). The plates on blood agar and chocolate-agar were incubated at 35° ± 2 °C. Daily readings of the plates were held up to 48 h.

### Preparation of drug treatment – *L. operculata* solution

**Figure 1:**
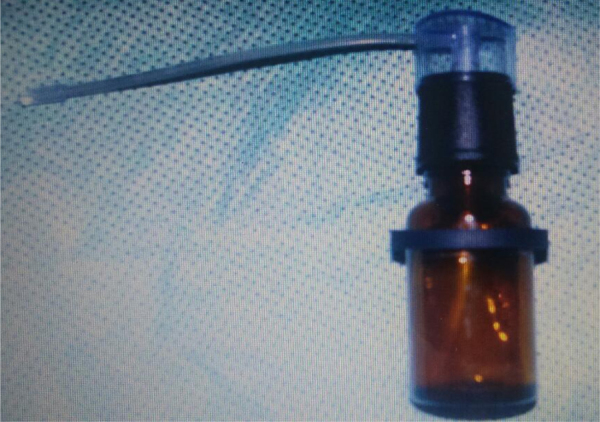
Atomizer used for drug application.

Using physiological saline solution as the solvent, dilution was prepared containing 0.1 g of *L. operculata* aqueous extract in 10 mL of saline solution to achieve the expected concentration. The solution was inserted into an atomizer (Fig. 1), which produced a jet of the mixture into a nozzle with resulting formation of microparticled aerosol. Each jet applied 0.5 mL of solution.

### Treatment

After RS induction period, animals in the therapeutic study group received treatment with nasal application of *L. operculata* aqueous extract diluted to 1% in saline solution. The animal head was hold vertically and the atomizer nozzle was inserted into the right nostril. The atomizer nozzle was pressed once. Then the device was cleaned and the process was repeated in the left nostril. The animals received a spray of the solution, three times daily throughout the treatment period. The control group received treatment with saline in the same form and amount of the study group for a period of 30 days. After five days of treatment, 20 animals from each group were sacrificed. The same procedure was repeated after 15 days and after 30 days.

### Histological evaluation of sinus mucosa

Immediately after sacrifice maxillary sinus lining mucosa samples were collected. The histologic parameters observed were inflammatory cells infiltration (infiltrate mild, moderate or severe), neovascularization (present or absent) and connective-fibrous proliferation (absent in isolated outbreaks or diffuse proliferation). All slides were evaluated by two different pathologists, blinded to the treatment protocol.

### Toxicity study

Sixty rabbits were divided into 3 groups receiving *L. operculata* solution at therapeutic concentration for a period of 30 days. After receiving the drug, the animals had blood samples, liver, kidney, brain and lung collected to histopathological evaluation.

### Statistical analysis

The values of mucosal histology were also described according to groups and times with the use of absolute and relative frequencies. They were compared using the nonparametric Wilcoxon test for the infiltration of inflammatory cells, connective, vascular and fibrous proliferation variables. Comparisons were performed to investigate the differences between groups or follow-up times. All tests were performed at 10% significance level.

## Results

Of the 180 animals started the experiment 8 died before the sacrifice time. Three of these animals belonged to study group, 2 to control group and 1 to toxicity group. Among these 8, 3 died during the induction RS period. The other animals died soon after the start of the treatment period.

One animal that belonged to study group died six days after the onset of administration of *L. operculata* nasal solution, 1 belonged to the control group and died between 2 and 6 days after the onset of nasal administration of physiologic solution. Of the 8 animals, 7 died from gastro-enterocolitis and 1 due to pneumonia. All the animals at the end of RS induction period had purulent rhinorrhea at the side where the sponge was placed and none of them had contralateral rhinorrhea.

In order to identify possible histological changes that could be related to the continuous use of the test substance we histologically evaluated by hematoxylin eosin the following organs: brain, heart, lung, kidney and liver. There were no abnormalities that could be related to drug use.

### Sinus secretion culture

Sinus secretions were taken from the inside of each rabbit maxillary sinus with swab (Cuturet^®^) after sacrifice. The secretions collected were harvested in blood agar and chocolate-agar. The bacteria found after the procedure are described in Table 1.

**Table 1:** Description of bacteria identified in the cultures of material collected in the nostrils of animals at the start of the treatment, control and study groups.

Microorganism	Blood agar	Chocolate agar
*Acinetobacter lwoffii*	16	3
*Acinetobacter baumanii*	1	–
*Alcaligenes* sp.	41	24
*Bacillus* sp.	7	20
*Bacillus subtillis*	3	2
*Micrococcus* sp.	–	1
Negative after 48 h incubation	8	7
*Escherichia coli*	1	–
*Pseudomonas* sp.	7	1
*Sphingomonas* sp.	1	1
*Staphylococcus aureus*	23	7
*Streptoccocus pyogenes*	8	45
*Staphylococcus coagulase negative*	3	3
*Streptococcus viridans*	1	2
*Pseudomonas aeruginosa*	–	2

### Histological findings

**Figure 2:**
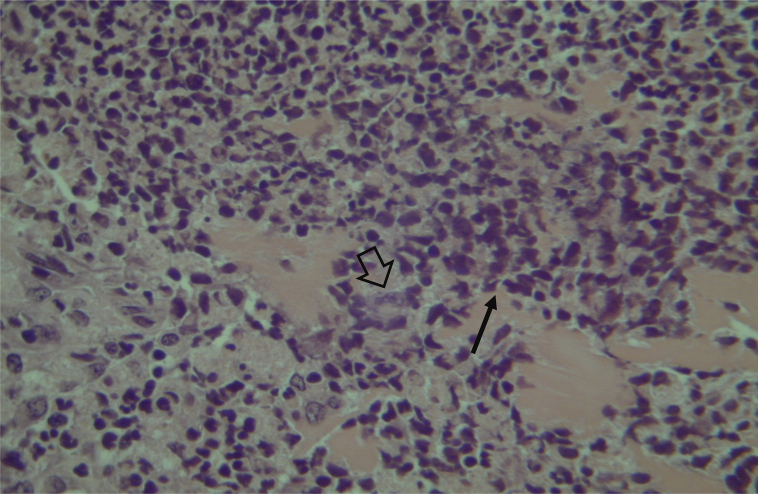
Mucosa of the maxillary sinus showing intense lymphocytic infiltrate (narrow arrow) and glandular destruction (wide arrow) – optical microscopy, HE staining, 200×.

**Figure 3:**
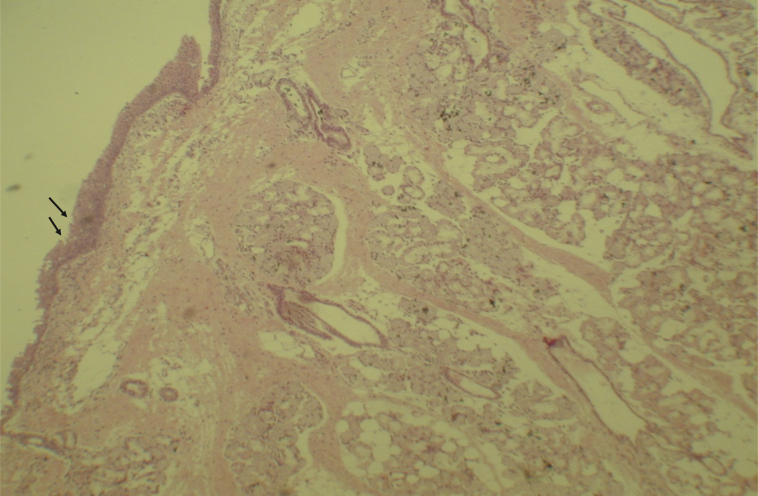
Mucosa of the maxillary sinus showing areas of erosion in the epithelium (arrows) and inflammatory cells. Optical microscopy, HE staining, 100×.

**Figure 4:**
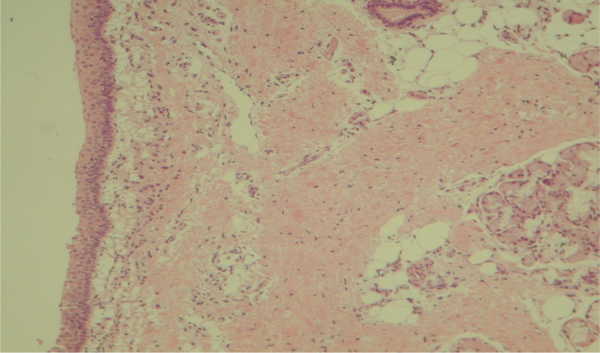
Mucosa of the maxillary sinus showing normal epithelium, without inflammatory process. Optical microscopy, HE staining, 100×.

Histological evaluation of sinus mucosa showed various degrees of inflammation, characterized from intense infiltration of inflammatory cells (Fig. 2), epithelial alterations (Fig. 3), neovascularization, glandular destruction and connective-fibrous proliferation to mucosa practically normal (Fig. 4). Such variations were present in both treatment groups (Table 2).

**Table 2:** Results of data comparisons between parameters–acute inflammation, neovascularization and fibrous connective proliferation for different treatment follow up.

Acute inflammation	*p*
Time 5	0.186299
Time 15	0.052137
Time 30	0.08863
General	0.08863

*Neovascularization*	
Time 5	1
Time 15	0.567169
Time 30	0.002849
General	0.002849

*Fibrous connective proliferation*	
Time 5	1
Time 15	1
Time 30	0.025582
General	0.025582

These data show a statistically significant difference between groups and treatment times in the overall assessment of the three different criteria used for histological analysis.

Histological studies showed no toxicity in the studied organs that could be related to the use of *L. operculata*.

### Chemical definition

Phytochemical assessment of *L. operculata* was done through liquid chromatography with reversed phase column. Elution was carried out in the gradient mode at a flow rate of 1.0 mL/min and UV detection at 254 nm. The major substance was chosen as phytochemical marker. The quantitative analysis was performed using the external standard method, and the marker, duly purified and identified, used as standard.

### Purification of phytochemical marker

Aqueous crude extract was fractionated by solid phase extraction on reverse phase (EFS-C18), using C18 silica gel as adsorbent. Ten fractions were obtained and then they were eluted with water initially and subsequently mixtures of water/MeOH to 100% MeOH. Final purification of the compound was carried out by HPLC.

### Structural elucidation of the marker

The NMR spectra were obtained on spectrometer 500 (11.7 T), at 500 MHz and 125 MHz for 13 C, with samples dissolved in D2O. High-resolution mass spectra were obtained and the spectra were obtained in positive and negative modes.

The purified substance obtained in the form of an off-white solid, was identified as 2,3-dicafeoilglicaric acid. This compound was not described previously in literature.

The spectrum of high-resolution mass in the positive mode presented cations at *m*/*z* 543.1177 [M+Na] + (calculated 543.1109) and negative mode showed the ion at *m*/*z* 519.1221 [MH] – (calculated 519.1144) indicating molecular formula C24H24O13. Furthermore, one can observe loss of two caffeoyl units (162) for MS2 in both the positive mode and in the negative mode. The chromatographic purity of this pattern was determined to be equal to 86.97%.

### In vitro evaluation

*Staphylococcus aureus* ATCC 25923–methicillin-susceptible (MSSA) and *Staphylococcus aureus* ATCC 43300–methicillin-resistant (MRSA) were used as bacterial subjects and cultivated on Muller-Hilton plates. After 24 h incubation it was observed bacterial growth on all samples. The plates were cultivated with and without the presence of *L. operculata* in aqueous extract 1% and 0.5% and 2,3-dicafeoilglicaric acid 1%. The higher the concentration of the drug, the greater was the inhibition of bacterial growth on the samples. When isolated, active principle proved to be more effective than the samples with *L. operculata* extract at the same concentrations. The active principle 1% was more effective than the 1% aqueous extract, which in turn was more efficient than 0.5% aqueous extract. All plates with *Streptococcus pyogenes* ATCC 19615 had their bacterial growth compromised by the action of *L. operculata* in different concentrations and no difference was observed for *Staphylococccus aureus* ATCC 25923–Methicillin-Susceptible (MSSA) and *Staphylococcus aureus* ATCC 43300–Methicillin-Resistant (MRSA).

## Discussion

Different studies have highlighted the importance of local treatment of sinus disease.^11^, ^12^ *L. operculata* is used to treat inflammatory diseases of the upper airway in homeopathic and allopathic medications produced in Europe, North America and Brazil.^13^, ^14^ Despite the widespread use of *L. operculata* there are few studies that attest to its therapeutic value.^15^

The exact mechanism of action of *L. operculatta* is still unclear. According to Matos and Gottlieb (1967),^16^ the active ingredient isocucurbitacin B presents biological activities with decongestants actions as well as laxative, hemolytic, embryo toxic and abortion inductive proprieties. Thus, in view of reports confirming the toxicity of cucurbit cines and it is assumed that the isocucurbitacin B is the toxic principle of *L. operculata*.^16^

After the establishment of effective experimental models and reproducible rhino sinusitis in rabbits, many authors have used this tool to study and compare various forms of treatment for nasal infection.^17^, ^18^, ^19^ Some authors even rated the herbal effect of local application in these situations.^20^

Some studies have already evaluated the histological inflammatory pattern of sinusitis in animal models.^21^, ^22^

Some of these studies have used experimental sinusitis models to compare different treatments. Some authors use the histological evaluation of the nasal or sinus mucosa as inflammation intensity parameter. In most cases, this analysis is performed qualitatively.^22^ However, in other studies, this analysis is also done by semi-quantitative technique.^21^ Therefore, they are often assessed factors such as infiltration of inflammatory cells, epithelial ulceration, cilliary loss, edema and connective-fibrous proliferation.^23^

In agreement with our study, other authors point out that the bacterial sinusitis induction technique is effective^19^, ^20^, ^21^, ^22^, ^23^ and the etiological agents of infection is related to the infection model used.^24^

The antimicrobial effect and secretive induction are probably the main activities of *L. operculata*. These actions are known to be important for treating different respiratory infections such as RS.^23^

Nasal topical administration *Luffa* extract solution operculata 1% has shown superior efficacy to the saline for the treatment of bacterial rhino sinusitis in experimental rabbit model, taking into account, histological and sinus secretions culture parameters.

The positive results observed in the in vivo test combined with irritant effect on the respiratory tract already described in literature as a side effect of *Luffa*, stimulated us to identify active ingredients in *Luffa* extract and submit them to antibacterial activity tests.

The purified substance obtained was identified as 2,3-dicafeoilglicaric acid. This compound was not described previously in literature. The substance proved to be effective in vitro tests inhibiting the growth of bacteria of the species *Streptococcus pyogenes*. Since the treatment of rhino sinusitis with topical aqueous extract of *L. operculata* has side symptoms due to the presence of saponins which cause some irritation to the airways, the use of the active principle in the absence thereof may prove to be of great value in the treatment of RS in humans, bringing the possibility of a new drug for topical use with great practical utility and commercial viability.

## Conclusion

Topical nasal solution of the aqueous extract of *L. operculata* is effective compared to the application of saline solution for the treatment of bacterial RS in an experimental model. *L. operculata* determined in vitro inhibition of growth of *S. pyogenes.*

## Conflicts of interest

The authors declare no conflicts of interest.
